# Polymyositis following Pandemic Influenza A (H1N1) and 2009-10 Seasonal Trivalent Vaccines

**DOI:** 10.1155/2012/836930

**Published:** 2012-08-15

**Authors:** Clodoveo Ferri, Michele Colaci, Carlo Umberto Manzini, Marco Sebastiani, Dilia Giuggioli, Lucio Brugioni

**Affiliations:** ^1^Rheumatology Unit, Azienda Policlinico-Universitaria Policlinico di Modena, Via del Pozzo 71, 41124 Modena, Italy; ^2^Rheumatology Unit, Department of Internal Medicine, University of Modena and Reggio Emilia and Azienda Ospedaliero-Universitaria Policlinico di Modena, Via del Pozzo 71, 41124 Modena, Italy; ^3^Critical Care Unit, Azienda Ospedaliero-Universitaria Policlinico di Modena, Modena, Italy

## Abstract

Sporadic associations between inflammatory myopathies with vaccinations were described in the literature, raising the possible trigger value of vaccines in the development of these autoimmune disorders. Here, we reported the clinical history of 3 patients who developed polymyositis complicated by interstitial lung disease (2 cases) and dermatomyositis (1 case), after influenza A (H1N1) vaccination.

## 1. Introduction

The safety of pandemic influenza A (H1N1) vaccine, given alone or simultaneously with seasonal trivalent influenza vaccine, has been demonstrated [1–5]. Vaccine-related adverse events are generally mild, including pain at injection site, fever, myalgias, and fatigue. Here, we described 3 patients, firstly referred to Infectious Disease Unit of our university-based hospital and successively to our Rheumatology Unit, because of recent-onset high fever, respiratory symptoms, and marked muscle weakness. This clinical picture followed the administration of pandemic A H1N1 (Focetria, Novartis) and seasonal trivalent (Vaxigrip, Sanofi Pasteur MSD) influenza vaccines. No previous infections before the vaccinations were reported. Patients' clinicoserological features are summarized in Table 1.

## 2. Case reports

### 2.1. Patient 1

In November 2009, this 59-year-old woman developed high fever, cough, and dyspnea few days after receiving influenza vaccines. Two weeks later, she was hospitalized because of the rapid worsening of symptoms, resistant to antibiotic therapy. Diffuse infiltrates in both lungs and marked proximal muscle weakness were observed; wide laboratory and radiological evaluations, including lung high-resolution computed tomography (HRCT) and bronchoalveolar lavage (BAL), excluded infectious or neoplastic diseases. In January 2010, diagnosis of polymyositis (PM) with diffuse organizing pneumonia was done on the basis of clinicoserological and EMG alterations. Patient's general conditions and muscle involvement rapidly improved since the first week of steroid treatment; however, considering that a second lung HRCT revealed an evolution to diffuse interstitial involvement, mycophenolate mofetil was added at the fifth week of steroid treatment. To date, PM remains slightly active (CK 150–200 UI/mL), along with a fibromyalgic/depressive syndrome.

### 2.2. Patient 2

This 60-year-old male patient presented a past history of recurrent episodes of cough, fever, and arthromyalgias. In December 2009, one month after influenza vaccines, he showed high fever, dyspnea, and rapidly progressive proximal muscle weakness. At the patient's hospitalization, bibasilar lung infiltrates were observed, followed by rapid increase of creatinine kinase. Possible infectious or neoplastic diseases were excluded by means of instrumental investigations, such as HRCT and BAL fluid examination; therefore, diagnosis of PM with basilar organizing pneumonia was done. Since the first 2 weeks of steroid treatment, the patient showed a rapid improvement of both muscle and lung involvements. Interestingly, patient 2 showed serum anti-ENA-Jo1 antibody, a typical immunological marker of PM complicated by interstitial lung disease [6]. Up to date, PM is well controlled with ongoing therapy; a fibromyalgic syndrome remains as a sequel.

### 2.3. Patient 3

In December 2009, this 71-year-old female patient presented proximal muscle weakness, severe dysphagia, periungueal erythema, and periocular bullous heliotropic lesions, just 7 days after the influenza vaccinations. The typical electromyographic alterations and the straight raise of CK confirmed the diagnosis of polydermatomyositis (DM). The patient started steroid treatment at dosage of 1 mg/kg/day, slowly tapered in the following period, along with monthly immunoglobulin's infusions until August 2010. In November 2010, despite the good response to therapy of myositis, the patient presented an abrupt deterioration of dermatitis, with appearance of generalized erythroderma; thus, steroid dosage was raised again to 1 mg/kg/day. After about 40 days, the patient died for an Epstein-Barr-related acute pneumonia.

## 3. Discussion

We presented 3 patients with inflammatory myopathies, associated with interstitial lung disease in 2 cases, following the vaccinations with H1N1 plus the seasonal trivalent influenza. Interestingly, 2/3 cases are Italian citizens with the genetic origin in sub-Saharan Western Africa; this finding is difficult to entirely explain, but it is consistent with known epidemiological data, which evidenced that inflammatory myositis is more common in Afro-Americans [7].

In the literature, just one case of dermatomyositis after the influenza vaccine was described by Jani et al. [8]. These authors reported the clinical history of a 68-year-old woman developing myositis with typical heliotrope discoloration over eyelid two weeks after the vaccination; the treatment with oral high-dose prednisolone and azathioprine was effective. Other sporadic case reports suggested the possible association between myopathies with vaccination for hepatitis B virus, mycobacterium tuberculosis, tetanus, smallpox, polio, diphtheria, and diphtheria-pertussis-tetanus [9]. Indeed, inflammatory myopathies are recognized to be triggered also by viral infections in predisposed individuals [9].

Recent studies on large series of individuals pointed out the safety of the 2009 pandemic influenza A H1N1 vaccine [4, 5]. However, possible cases of inflammatory myopathies triggered by this vaccination might have not been recorded in these studies; in fact, (i) clinical signs might be developed after few weeks from vaccines; (ii) possibly, the diagnosis of PM/DM may be formulated long time later or overlooked entirely. Therefore, it is supposable that the actual incidence of autoimmune disorders after influenza vaccination can be underestimated in general clinical practice. On the other hand, we cannot definitely exclude that the systemic manifestations following influenza vaccination in our three patients may be coincidental; however, it is supposable that vaccine-related viral antigens and/or adjuvants might play a triggering role responsible for the observed autoimmune complications, possibly in genetically predisposed subjects. This hypothesis is in keeping with numerous clinical observations suggesting a possible link between myopathies and infectious triggering agents [8–10].

## Figures and Tables

**Table 1:** 

	Patient 1	Patient 2	Patient 3
Sex/age (y)/race/smoke	F/59/Afro-American/—	M/60/African/—	F/71/Caucasian/—

Symptoms	Fever, cough, dyspnea, diffuse lung infiltrates, proximal muscle weakness	Fever, cough, dyspnea, basilar lung infiltrates, proximal muscle weakness	Proximal muscle weakness, severe dysphagia, periungueal erythema, periocular bullous heliotropic lesions
Onset after vaccination	5 days	30 days	7 days

CK (nv < 170 U/L) 1-2-3	2,685-569-98	6,589-1,700-752	1,325-67-20

EMG myogenic alterations	+	+	+

Autoimmunity	ANA 1 : 640 (speckled); ENA- ASMA-; AMA-; ANCA-	ANA 1 : 320 (speckled); ENA+ (Jo1); ASMA+ 1 : 160; AMA-; ANCA-	ANA 1 : 320 (speckled); ENA+ (Ro/SSA); AMA-; ASMA-; ANCA-

Treatment	6-MP 1 mg/Kg/day → slowly tapered to 8 mg/day; Mycophenolate mofetil 2 g/day from February 2010	Predn. 1.5 mg/Kg/day → slowly tapered to 25 mg/day Methotrexate 20 mg/week; IVIG 1 mg/kg monthly (February–May 2010); start of Cyclosporine 200 mg/day from September 2010	6-MP 1 mg/Kg/day → slowly tapered to 16 mg/day; IVIG 1 mg/kg monthly (March–August 2010)

Outcome	Remission	Partially improved	Died in November 2010 (Epstein-Barr virus reactivation)

CK: creatinine kinase (1: at baseline; 2: 1 month after therapy beginning; 3: at the end of followup, in December 2010); EMG: electromyography; ANA: antinuclear antibodies; anti-ENA: antiextractable nuclear antigen antibodies; ASMA: antismooth muscle antibodies; AMA: antimitochondrial antibodies; ANCA: antineutrophil cytoplasmic antibodies; 6-MP: 6-methylprednisolone, Predn: prednisone.
